# Exosomes May Be the Potential New Direction of Research in Osteoarthritis Management

**DOI:** 10.1155/2019/7695768

**Published:** 2019-11-03

**Authors:** Cheng Ju, Renfeng Liu, Yu Zhang, Feifei Zhang, Jun Sun, Xiao-Bin Lv, Zhiping Zhang

**Affiliations:** ^1^Jiangxi Key Laboratory of Cancer Metastasis and Precision Treatment, The Third Affiliated Hospital of Nanchang University, Nanchang, China; ^2^Department of Orthopedics, The Third Affiliated Hospital of Nanchang University, Nanchang, Jiangxi 330008, China; ^3^Nanchang Key Laboratory of Orthopaedics, The Third Affiliated Hospital of Nanchang University, Nanchang, China; ^4^Medical Department of Graduate School, Nanchang University, Nanchang, Jiangxi 330006, China

## Abstract

Osteoarthritis (OA) is a joint degenerative disease, which is prominent in the middle-aged and elderly population, often leading to repeated pain in the joints of patients and seriously affecting the life quality of patients. At present, the treatment of OA mainly depends on the surgery and drug treatment. Nevertheless, these treatments still face many problems, such as surgical safety, complications, and drug side effects. Exosomes can be secreted and released by multiple cell types and have lipid bilayer membranes and contain abundant biological molecules, including proteins, lipids, and nucleic acids. Moreover, exosomes play a critical role in local and distal intercellular and intracellular communication. In recent years, several studies have found that exosomes can regulate the progression of OA and have a potential efficacy for OA treatment. Thus, in this article, we summarize and review the relevant research of exosomes in OA and emphasize the importance of exosomes in the development of OA.

## 1. Introduction

OA is a clinical disease involving the entire joint, including cartilage, subchondral bone, meniscus, ligaments, and muscles [1]. The main onset of OA is in the middle-aged and elderly population, which is characterized by cartilage degradation, subchondral bone sclerosis, osteophyte formation, synovial inflammation, and angiogenesis, and key risk factors include trauma, age, obesity, genetic and joint damage, and metabolic diseases [1–4]. At present, surgical treatment and drugs have achieved certain effects in patients with OA, but the accompanying surgical safety and complications and drug side effects are still clinical problems [5]. In addition, OA has caused huge public health resources [6]. Therefore, it is urgent to explore new strategies to protect cartilage and delay OA development.

Exosomes are an extracellular vesicle (EV) that functions as signaling molecules between cells [7]. In present studies, exosomes are closely related to OA, including promoting cartilage formation and tissue repair and regulating inflammatory response and homeostasis [8–10]. These studies have revealed the significant role of exosomes in OA. Besides, the clinical application of exosomes has received extensive attention. Therefore, it is very important to explore the role of exosomes in the OA process. In this review, we describe the characteristics of exosomes, biological functions, and related studies in osteoarthritis. Also, we discuss the diagnostic value and therapeutic potential of exosomes in OA.

## 2. Exosomes: An Overview

**Figure 1:**
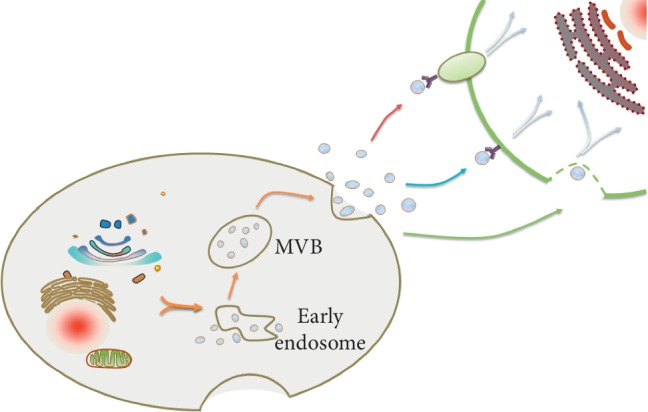
Exosomal formation, release, and intercellular transmission. The formation of exosomes includes three stages: form endosomes, form ILVs and MVBs, and further are secreted to extracellular space. When exosomes are released, exosomes act as intercellular signaling molecules that interact with receptor cells, including binding to cell membrane surface proteins, direct fusion, and endocytosis.

EVs were divided into microvesicles, exosomes, and apoptotic bodies in accordance with morphological features and content [11]. The most studied are the exosomes, which are a kind of EV and can be secreted and released by multiple cell types (usually, exosomal size ranging from 30 to 150 nm) [12]. Exosomes were first found by R. M. Johnstone et al. when studying the formation of vesicles during the process of mature reticulocytes in sheep. Exosomes were widely distributed in peripheral blood, urine, saliva, ascites, milk, cerebrospinal fluid, and other body fluids and were usually extracted by ultracentrifugation, filtration centrifugation, density gradient centrifugation, chromatography, immunomagnetic beads, and polyethylene glycol precipitation [13, 14]. More importantly, the isolation and identification of high-purity exosomes are essential for elucidating its mechanism of action and its biological function. At present, a variety of techniques have been used to identify exosomes, including nanoparticle tracking analysis (NTA), dynamic light scattering (DLS), resistive pulse sensing, flow cytometry, electron microscopy, and atomic force microscopy (AFM) [15]. In general, the formation of exosomes involves three stages (Figure 1): (a) involution of cell membranes to form endosomes; (b) the endosomal membrane sprouts inward to form intraluminal vesicles (ILVs), and the endosomal body becomes multivesicular body biogenesis (MVBs); (c) exosomes are secreted to extracellular space by fusion of MVBs and plasma membrane [16]; extracellular exosomes have lipid bilayer membranes and contain abundant biological molecules, including proteins, lipids, and nucleic acids [17]. Since the first discovery of sheep erythrocyte supernatant, exosomes have been extensively studied and found to be closely associated with the occurrence and development of various diseases, including cancer, immune diseases, and neurodegenerative diseases [18–20]. The critical reason is that exosomes play a key role in local and distal intercellular and intracellular communication [21]. Exosomes are known to have a wide range of biological functions, such as regulating angiogenesis, apoptosis, antigen presentation, and receptor-mediated endocytosis. In addition, exosome can be used as a biomarker for the diagnosis and prognosis of the disease and shows great clinical value [22].

## 3. Exosome-Derived miRNAs in OA

Exosomes contain various RNAs including miRNA, lncRNA, circRNA, and mRNA. miRNAs are a kind of tiny, highly conserved RNAs with a length of about 22 nucleotides [23]. miRNAs have been studied for more than ten years in noncoding RNA. In the past years, miRNAs were extensively studied in diverse disciplines. With the in-depth study, the biological function of miRNAs has been gradually clarified. Critically, miRNAs contain miRNA response element, which can complement downstream target genes and degrade them [24]. This is an important function of miRNA.

Mesenchymal stem cells (MSCs) are derived from mesodermal cells and have the ability to differentiate into many kinds of cells, including osteogenic differentiation, adipogenic differentiation, and chondrogenic differentiation [25]. In addition, MSCs had better immune tolerance and immune regulation. These advantages are used to incorporate a new strategy to control the intervention of inflammation. In recent years, the studies and treatment of mesenchyme in OA have been extensively reported. MSCs have shown important functions in cartilage repair and inflammation regulation of osteoarthritis in some studies [26, 27]. Further, exosomes driven from MSCs have recently attracted widespread attention. For example, in the infrapatellar fat pad (IPFP) MSC-derived exosomes (MSCIPFP-Exos), miR-100-5p regulates MSCIPFP-Exos-mediated protection of articular cartilage via the mTOR signaling pathway [28]. After TGF-*β*1 induced MSCs, MSC-exosome-derived miR-135b was significantly increased in chondrocyte and overexpression of mir-135b promoted cartilage proliferation and repair. In the mechanism, miR-135b can bind with the transcription factor, specificity protein 1 (SP1), which is a protein that regulates apoptosis and proliferation of cells, targeting the degradation of SP1 to promote cartilage proliferation [29]. The procartilage repair effect of miR-135b has also been confirmed in the SD rat inflammation model. Similarly, through the verification of cell levels and animal models, the MSC-exosome-derived miR-92a-3p/wnt5a axis has a significant effect in increasing cartilage proliferation, slowing the progression of OA, maintaining cartilage stability, and inhibiting cartilage degradation [30]. Moreover, human synovial mesenchymal stem cell (hSMSC)-derived miR-140-5p can delay the progression of early OA and prevent knee cartilage damage, but the effect of hSMSC-derived miR-140-5p is finite [31]. On the other hand, miRNA-95-5p is significantly expressed in normal human cartilage by miRNA microarray analysis of normal cells and OA chondrocytes in human chondrocyte-derived exosomes. Exogenously derived miRNA-95-5p overexpression can delay the progression of OA and maintain cartilage development and homeostasis. Mechanistically, miRNA-95-5p-HDAC2/8 pathway plays a key role [9]. Therefore, these studies bring a potential new approach to the prevention and treatment of OA.

In the above studies, miRNAs are closely related to cartilage proliferation, slowing the progression of OA, maintaining cartilage stability, and inhibiting cartilage degradation. To elucidate the specific mechanism of action of exosome-derived miRNAs has provided a guiding direction for OA treatment in the future.

## 4. Exosome-Derived lncRNAs in OA

Lncrna is a class of RNA that is more than 200 nt in length and does not encode proteins [32]. lncRNA exists in the nucleus or cytoplasm, and its function depends on the location of the subcellular [33]. Diverse lncRNA expression can regulate the cell proliferation, migration, invasion, immunobiology, and differentiation [34, 35]. In addition, lncRNA can perform its biological functions through different mechanisms, including decoys, signals, guides, and scaffolds [36]. In recent years, lncRNA is closely associated with the progression of OA and a variety of lncRNAs have been found to regulate the proliferation, repair, and formation of articular cartilage [37, 38]. The classical pathway of lncRNA is that lncRNA acts as competitive endogenous RNA (ceRNA) by binding to specific miRNAs [35]. In previous studies, MSC-Exos have the function of improving cartilage and bone regeneration [39, 40]. In a study by Liu et al., exosomal KLF3-AS1 derived from MSCs exhibits significant promotion of cartilage growth and reduced inflammation-induced cartilage damage. Among them, lncRNA KLF3-AS1 plays an important role in promoting GIT1 expression by sponge to miR-206 as a ceRNA [41].

### 4.1. Exosome-Derived lncRNA as Molecular Markers

Clinically, the diagnosis of early and advanced osteoarthritis lacks simple and accurate marker detection and the identification and utilization of a molecular marker as a specific clinical value of staging diagnosis of OA. In a study by Zhao and Xu, the subjects were divided into three groups: the control group, the early OA group, and the late OA group (patients in the late-stage OA). Blood samples from the elbow vein and synovial fluid samples from the knee joint were collected from all subjects. Exosomes were extracted by ultracentrifugation, and the expression of several exosomal lncRNAs was measured using RT-PCR. With the development of OA, the expression of exosomal lncRNA PCGEM1 is gradually increasing. This shows that exosome-derived lncRNA can be an important indicator of the progression of osteoarthritis and provides a new molecular marker for the accurate and effective monitoring of the progress of osteoarthritis [42]. Numerous molecules in exosomes play an important role in regulating the development of OA and detecting the progress of OA, and we list all OA-related exosome-derived miRNAs and lncRNAs in Table 1.

## 5. Exosomes Regulate the Progression of OA

### 5.1. Exosomes Regulate Cellular Senescence of OA

Aging is a key factor for inducing the progression of OA, and age-related proinflammatory state may act as a vital role in OA [43]. In addition, OA cartilage can be detected by the senescence-associated secretory phenotype (SASP) and oxidative stress is a key process in inducing cellular senescence [44, 45]. Researchers found that EV derived from adipose-derived mesenchymal stem cells (ADMSCs) reduces inflammation and oxidative stress. These data show that conditioned medium (CM) and exosome mediate antisenescence effects in OA osteoblasts. Of note, osteoblasts participate in the regulation of cartilage metabolism and bone remodeling in OA [46]. Therefore, regulation of OA cellular senescence and metabolism provides new targets for the prevention or treatment of OA.

### 5.2. Exosomes Regulate Cartilage Development and Homeostasis

In the rat model, for rats with large segmental cartilage defects, the function of cartilage was detected by intra-articular injection of embryonic mesenchyme-derived exosomes. After 12 weeks of treatment, the study showed that defects in treated exosomes revealed cartilage and complete recovery of subchondral bone characterized by hyaline cartilage with good surface regularity, complete binding of adjacent cartilage, and extracellular matrix deposition very similar to age-matched unoperated controls. In contrast, in the control group treated with PBS, only fibrous repair tissue was found in the cartilage defect [39]. This research first discovered the effectiveness of exosomes for cartilage repair. Next, Wang et al. showed in mouse OA model that the injection of embryonic stem cell-derived exosomes into the joint cavity can slow the progression of OA and maintain the cartilage matrix [8]. However, MSC-derived exosomes effectively relieve temporomandibular joint osteoarthritis (TMJ-OA) pain and promote regeneration [47]. These studies provide a feasible solution for the clinical treatment of cartilage defects and repair of cartilage, indicating that the new prospect of exosome treatment as OA is worthy of expectation. The repair effect of exosomes in OA is significant. Is the effect of exosomes from different sources on OA different? In one study, the authors compared the effects of exosomes from induced pluripotent stem cell-derived MSCs (iMSCs) and synovial membrane mesenchymal stem cells (SMMSCs) in the mice OA model. The study found that iMSC-Exos has a better therapeutic effect after the same injection of iMSC-Exos or SMMSC-Exos. Furthermore, although both iMSC-Exos and SMMSC-Exos stimulate chondrocyte migration and proliferation, iMSC-Exos has a greater effect than SMMSC-Exos [48].

### 5.3. Exosomes Regulate Inflammation

Synovial membrane is required for normal cartilage and joint function. Synovial inflammation (synovitis) plays a key role in the development of symptoms and progression of OA, and a model of Toll-like receptor and complement activation as an important mechanism contributes to the synovitis and enhances cartilage erosion in OA [49]. On the one hand, inflammation can stimulate angiogenesis, and on the other hand, angiogenesis can promote inflammation. Furthermore, angiogenesis also promotes chondrocyte hypertrophy and endochondral ossification [50].

**Figure 2:**
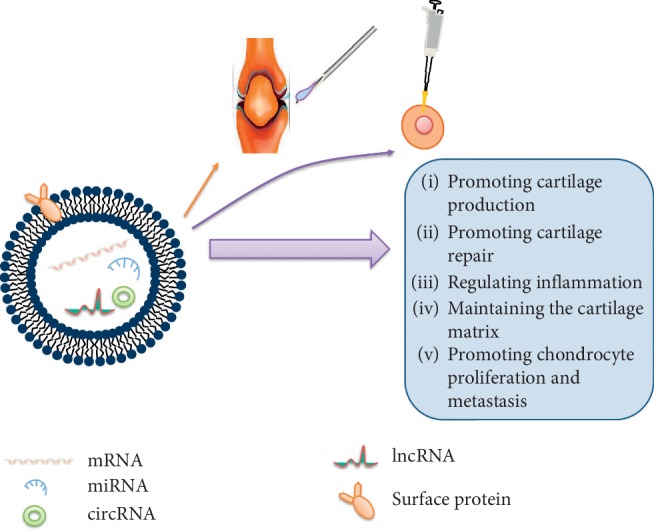
The function of exosomes in OA. We describe the important functions of exosomes in OA through intra-articular injection and cell transfection, including promoting cartilage production, promoting cartilage repair, regulating inflammation, maintaining the cartilage matrix, and promoting chondrocyte proliferation and metastasis. These indicate the potential clinical value of exosomes for OA treatment.

In the above description, exosomes exhibit a satisfactory effect on inflammation inhibition and protection of cartilage. Conversely, in synovial-derived exosomes, proinflammatory action is remarkable after treatment of macrophages with exosomes, and exosomes also promote the release of various chemokines and metalloproteinases (MMPs) [51]. IL-1*β* is a key factor in cartilage degradation and joint inflammation [52]. The precise mechanism of exosomes involved in the pathogenesis of OA remains unclear, so further elucidation of the mechanism is important for our effective monitoring and treatment of OA. Finally, we describe the function of exosomes in OA in Figure 2.

## 6. Prospect of Clinical Application

Cell transplantation technology has shown great clinical potential as a new treatment. Therapeutic purposes are achieved by the local transplantation of autologous or allogeneic stem cells in vivo. MSCs have great anti-inflammatory and tissue repair effects [53]. In preceding studies, MSC has an excellent effect on heart disease, kidney disease, lung disease, nervous system disease, bone injury, and OA [54–59]. In addition, in the clinical trials of osteoarthritis, joint injection therapy of mesenchymal stem cells has a better therapeutic effect [60, 61]. There are still serious problems in immune rejection and ethics when MSCs are transplanted into damaged organisms [62]. In addition, economic, regenerative, and security issues remain issues of concern [53]. Of note, it is generally believed that the paracrine function of mesenchymal stem cells plays a major role, and its secretions have strong immunoregulatory effects, such as extracellular vesicles, exosomes, and cytokines, of which exosomes is an important substance [63]. Exosomes, as a secreted extracellular vesicle, have good intercellular communication function and no cell structure, which dramatically reduce immune rejection and have good safety and efficacy. As a safe and effective treatment, exosomes can avoid the problems of transfer of infectious pathogens, genetic instability, and malignant transformation of damaged sites [64]. In previous reports, exosomes have an observable effect on cartilage repair and delayed degradation in basic research and animal experiments. Therefore, development and application of exosome therapy as a therapeutic agent in regenerative medicine have good clinical application prospects.

## 7. Discussion

Good effects of exosomes have been shown in many diseases. However, the problems such as how to increase the secretion of exosomes more efficiently, whether it can promote the transport of exosomes to target cells through specific conditions, whether it can fully exert its repairing effect by controlling the pH of the microenvironment of its action, and whether the exocrine secreted by the tissue has different effects on the repair effect of the damage make the exosomes actually face many difficulties in clinical application. In OA patients, surgical treatment poses a definite safety risk and causes greater pain to the patient. The treatment of drugs also has certain side effects. Therefore, safe, effective, and non-side-effect treatment modes are clinically explored. In addition, it is also essential for the prevention of OA diseases. With the deepening of research on exosome mechanisms and the continuous maturity of technical means, it is of high clinical value to operate exosome therapy in the prevention and treatment of OA. This is beneficial for OA patients to relieve pain and improve their quality of life.

## Figures and Tables

**Table 1:** Exosome-derived miRNAs and lncRNAs in OA.

Names	Function	Mechanism	Ref
miR-100-5p	Maintains cartilage homeostasis	mTOR signaling pathway	[28]
miR-135b	Promotes chondrocyte proliferation and cartilage repair	miR-135b/SP1	[29]
miR-92a-3p	Regulates cartilage development and homeostasis	miR-92a-3p/Wnt5a	[30]
miRNA-95-5p	Regulates cartilage development and homeostasis	miRNA-95-5p-HDAC2/8	[9]
miR-140-5p	Enhances proliferation and migration of chondrocytes	miR-140-5p/RALA	[31]
KLF3-AS1	Promotes cartilage repair and chondrocyte proliferation	KLF3-AS1-miR-206/GIT1 axis	[41]
PCGEM1	As a biomarker	No mention	[42]

## References

[1] Loeser R. F., Goldring S. R., Scanzello C. R., Goldring M. B. (2012). Osteoarthritis: a disease of the joint as an organ. *Arthritis & Rheumatism*.

[2] Palazzo C., Ravaud J. F., Papelard A., Ravaud P., Poiraudeau S. (2014). The burden of musculoskeletal conditions. *PLoS One*.

[3] Lotz M. K., Kraus V. B. (2010). New developments in osteoarthritis. Posttraumatic osteoarthritis: pathogenesis and pharmacological treatment options. *Arthritis Research & Therapy*.

[4] Glyn-Jones S., Palmer A. J. R., Agricola R. (2015). Osteoarthritis. *The Lancet*.

[5] Dieppe P., Lim K., Lohmander S. (2011). Who should have knee joint replacement surgery for osteoarthritis?. *International Journal of Rheumatic Diseases*.

[6] Kiadaliri A. A., Lohmander L. S., Moradi-Lakeh M., Petersson I. F., Englund M. (2018). High and rising burden of hip and knee osteoarthritis in the Nordic region, 1990–2015. *Acta Orthopaedica*.

[7] Théry C., Zitvogel L., Amigorena S. (2002). Exosomes: composition, biogenesis and function. *Nature Reviews Immunology*.

[8] Wang Y., Yu D., Liu Z. (2017). Exosomes from embryonic mesenchymal stem cells alleviate osteoarthritis through balancing synthesis and degradation of cartilage extracellular matrix. *Stem Cell Research & Therapy*.

[9] Mao G., Hu S., Zhang Z. (2018). Exosomal miR-95-5p regulates chondrogenesis and cartilage degradation via histone deacetylase 2/8. *Journal of Cellular and Molecular Medicine*.

[10] Kato T., Miyaki S., Ishitobi H. (2014). Exosomes from IL-1*β* stimulated synovial fibroblasts induce osteoarthritic changes in articular chondrocytes. *Arthritis Research & Therapy*.

[11] György B., Szabó T. G., Pásztói M. (2011). Membrane vesicles, current state-of-the-art: emerging role of extracellular vesicles. *Cellular and Molecular Life Sciences*.

[12] Simons M., Raposo G. (2009). Exosomes—vesicular carriers for intercellular communication. *Current Opinion in Cell Biology*.

[13] Foers A. D., Chatfield S., Dagley L. F. (2018). Enrichment of extracellular vesicles from human synovial fluid using size exclusion chromatography. *Journal of Extracellular Vesicles*.

[14] Xu R., Greening D. W., Zhu H.-J., Takahashi N., Simpson R. J. (2016). Extracellular vesicle isolation and characterization: toward clinical application. *Journal of Clinical Investigation*.

[15] Gurunathan S., Kang M.-H., Jeyaraj M., Qasim M., Kim J.-H. (2019). Review of the isolation, characterization, biological function, and multifarious therapeutic approaches of exosomes. *Cells*.

[16] Ha D., Yang N., Nadithe V. (2016). Exosomes as therapeutic drug carriers and delivery vehicles across biological membranes: current perspectives and future challenges. *Acta Pharmaceutica Sinica B*.

[17] Qin J., Xu Q. (2014). Functions and application of exosomes. *Acta Poloniae Pharmaceutica*.

[18] Tai Y.-L., Chen K.-C., Hsieh J.-T., Shen T.-L. (2018). Exosomes in cancer development and clinical applications. *Cancer Science*.

[19] Raposo G., Stoorvogel W. (2013). Extracellular vesicles: exosomes, microvesicles, and friends. *The Journal of Cell Biology*.

[20] Raposo G., Nijman H. W., Stoorvogel W. (1996). B lymphocytes secrete antigen-presenting vesicles. *Journal of Experimental Medicine*.

[21] Zhao H., Achreja A., Iessi E. (2018). The key role of extracellular vesicles in the metastatic process. *Biochimica et Biophysica Acta (BBA)—Reviews on Cancer*.

[22] Lin J., Li J., Huang B. (2015). Exosomes: novel biomarkers for clinical diagnosis. *The Scientific World Journal*.

[23] D’Adamo S., Cetrullo S. (2017). MicroRNAs and autophagy: fine players in the control of chondrocyte homeostatic activities in osteoarthritis. *Oxidative Medicine and Cellular Longevity*.

[24] Garofalo M., Condorelli G., Croce C. (2008). MicroRNAs in diseases and drug response. *Current Opinion in Pharmacology*.

[25] Kode J. A., Mukherjee S., Joglekar M. V., Hardikar A. A. (2009). Mesenchymal stem cells: immunobiology and role in immunomodulation and tissue regeneration. *Cytotherapy*.

[26] Kim C., Keating A. (2019). Cell therapy for knee osteoarthritis: mesenchymal stromal cells. *Gerontology*.

[27] Al-Najar M., Khalil H., Al-Ajlouni J. (2017). Intra-articular injection of expanded autologous bone marrow mesenchymal cells in moderate and severe knee osteoarthritis is safe: a phase I/II study. *Journal of Orthopaedic Surgery*.

[28] Wu J., Kuang L., Chen C. (2019). miR-100-5p-abundant exosomes derived from infrapatellar fat pad MSCs protect articular cartilage and ameliorate gait abnormalities via inhibition of mTOR in osteoarthritis. *Biomaterials*.

[29] Wang R., Xu B., Xu H. (2018). TGF-beta1 promoted chondrocyte proliferation by regulating Sp1 through MSC-exosomes derived miR-135b. *Cell Cycle*.

[30] Mao G., Zhang Z., Hu S. (2018). Exosomes derived from miR-92a-3p-overexpressing human mesenchymal stem cells enhance chondrogenesis and suppress cartilage degradation via targeting WNT5A. *Stem Cell Research & Therapy*.

[31] Tao S.-C., Yuan T., Zhang Y.-L., Yin W.-J., Guo S.-C., Zhang C.-Q. (2017). Exosomes derived from miR-140-5p-overexpressing human synovial mesenchymal stem cells enhance cartilage tissue regeneration and prevent osteoarthritis of the knee in a rat model. *Theranostics*.

[32] Kopp F., Mendell J. T. (2018). Functional classification and experimental dissection of long noncoding RNAs. *Cell*.

[33] Chen L.-L. (2016). Linking long noncoding RNA localization and function. *Trends in Biochemical Sciences*.

[34] Hung T., Wang Y., Lin M. F. (2011). Extensive and coordinated transcription of noncoding RNAs within cell-cycle promoters. *Nature Genetics*.

[35] Fatica A., Bozzoni I. (2014). Long non-coding RNAs: new players in cell differentiation and development. *Nature Reviews Genetics*.

[36] Ju C., Liu R., Zhang Y.-W. (2019). Mesenchymal stem cell-associated lncRNA in osteogenic differentiation. *Biomedicine & Pharmacotherapy*.

[37] Jiang S.-D., Lu J., Deng Z.-H., Li Y.-S., Lei G.-H. (2017). Long noncoding RNAs in osteoarthritis. *Joint Bone Spine*.

[38] Cen X., Huang X. Q., Sun W. T., Liu Q., Liu J. (2017). Long noncoding RNAs: a new regulatory code in osteoarthritis. *American Journal of Translational Research*.

[39] Zhang S., Chu W. C., Lai R. C., Lim S. K., Hui J. H. P., Toh W. S. (2016). Exosomes derived from human embryonic mesenchymal stem cells promote osteochondral regeneration. *Osteoarthritis and Cartilage*.

[40] Qi X., Zhang J., Yuan H. (2016). Exosomes secreted by human-induced pluripotent stem cell-derived mesenchymal stem cells repair critical-sized bone defects through enhanced angiogenesis and osteogenesis in osteoporotic rats. *International Journal of Biological Sciences*.

[41] Liu Y., Lin L., Zou R., Wen C., Wang Z., Lin F. (2018). MSC-derived exosomes promote proliferation and inhibit apoptosis of chondrocytes via lncRNA-KLF3-AS1/miR-206/GIT1 axis in osteoarthritis. *Cell Cycle*.

[42] Zhao Y., Xu J. (2018). Synovial fluid-derived exosomal lncRNA PCGEM1 as biomarker for the different stages of osteoarthritis. *International Orthopaedics*.

[43] Greene M. A., Loeser R. F. (2015). Aging-related inflammation in osteoarthritis. *Osteoarthritis and Cartilage*.

[44] Price J. S., Waters J. G., Darrah C. (2002). The role of chondrocyte senescence in osteoarthritis. *Aging Cell*.

[45] Itahana K., Campisi J., Dimri G. P. (2004). Mechanisms of cellular senescence in human and mouse cells. *Biogerontology*.

[46] Tofino-Vian M., Guillén M. I., del Caz M. D. P., Tofino-Castejón M. A., Alcaraz M. J. (2017). Extracellular vesicles from adipose-derived mesenchymal stem cells downregulate senescence features in osteoarthritic osteoblasts. *Oxidative Medicine and Cellular Longevity*.

[47] Zhang S., Teo K. Y. W., Chuah S. J., Lai R. C., Lim S. K., Toh W. S. (2019). MSC exosomes alleviate temporomandibular joint osteoarthritis by attenuating inflammation and restoring matrix homeostasis. *Biomaterials*.

[48] Zhu Y., Wang Y., Zhao B. (2017). Comparison of exosomes secreted by induced pluripotent stem cell-derived mesenchymal stem cells and synovial membrane-derived mesenchymal stem cells for the treatment of osteoarthritis. *Stem Cell Research & Therapy*.

[49] Scanzello C. R., Goldring S. R. (2012). The role of synovitis in osteoarthritis pathogenesis. *Bone*.

[50] Bonnet C. S., Walsh D. A. (2005). Osteoarthritis, angiogenesis and inflammation. *Rheumatology*.

[51] Domenis R., Zanutel R., Caponnetto F. (2017). Characterization of the proinflammatory profile of synovial fluid-derived exosomes of patients with osteoarthritis. *Mediators of Inflammation*.

[52] Goldring M. B., Otero M. (2011). Inflammation in osteoarthritis. *Current Opinion in Rheumatology*.

[53] Zorzopulos J., Opal S. M., Hernando-Insúa A. (2017). Immunomodulatory oligonucleotide IMT504: effects on mesenchymal stem cells as a first-in-class immunoprotective/immunoregenerative therapy. *World Journal of Stem Cells*.

[54] Hu H., Zou C. (2017). Mesenchymal stem cells in renal ischemia-reperfusion injury: biological and therapeutic perspectives. *Current Stem Cell Research & Therapy*.

[55] McGonagle D., Baboolal T. G., Jones E. (2017). Native joint-resident mesenchymal stem cells for cartilage repair in osteoarthritis. *Nature Reviews Rheumatology*.

[56] Mora A. L., Rojas M. (2013). Adult stem cells for chronic lung diseases. *Respirology*.

[57] Rosset P., Deschaseaux F., Layrolle P. (2014). Cell therapy for bone repair. *Orthopaedics & Traumatology: Surgery & Research*.

[58] Xiao J., Yang R., Biswas S., Qin X., Zhang M., Deng W. (2015). Mesenchymal stem cells and induced pluripotent stem cells as therapies for multiple sclerosis. *International Journal of Molecular Sciences*.

[59] Xu B., Luo Y., Liu Y., Li B.-Y., Wang Y. (2015). Platelet-derived growth factor-BB enhances MSC-mediated cardioprotection via suppression of miR-320 expression. *American Journal of Physiology-Heart and Circulatory Physiology*.

[60] Gao K., Zhu W., Li H. (2019). Association between cytokines and exosomes in synovial fluid of individuals with knee osteoarthritis. *Modern Rheumatology*.

[61] Lee W. S., Kim H. J., Kim K. I., Kim G. B., Jin W. (2019). Intra-articular injection of autologous adipose tissue-derived mesenchymal stem cells for the treatment of knee osteoarthritis: a phase IIb, randomized, placebo-controlled clinical trial. *STEM CELLS Translational Medicine*.

[62] Okano H., Nakamura M., Yoshida K. (2013). Steps toward safe cell therapy using induced pluripotent stem cells. *Circulation Research*.

[63] Lai R. C., Yeo R. W. Y., Lim S. K. (2015). Mesenchymal stem cell exosomes. *Seminars in Cell & Developmental Biology*.

[64] Aliotta J. M., Pereira N., Li M. (2012). Stable cell fate changes in marrow cells induced by lung-derived microvesicles. *Journal of Extracellular Vesicles*.

